# Using Untargeted LC-MS Metabolomics to Identify the Association of Biomarkers in Cattle Feces with Marbling Standard Longissimus Lumborum

**DOI:** 10.3390/ani12172243

**Published:** 2022-08-30

**Authors:** Dong Chen, Minchao Su, He Zhu, Gang Zhong, Xiaoyan Wang, Weimin Ma, Metha Wanapat, Zhiliang Tan

**Affiliations:** 1 College of Animal Science and Technology, Hunan Agricultural University, Changsha 410128, China; 2 College of Food Science and Engineering, Shandong Agriculture and Engineering University, Jinan 250100, China; 3 National Engineering Laboratory for Rice and By-Products Further Processing, College of Food Science and Engineering, Central South University of Forestry & Technology, Changsha 410004, China; 4 Technology Center of Gaoqing Black Cattle Product Processing and Quality Improvement, Zibo 255000, China; 5 Tropical Feed Resources Research and Development Center (TROFREC), Department of Animal Science, Facully of Agriculture, Khon Kaen University, Khon Kaen 40002, Thailand; 6 Institute of Subtropical Agriculture of the Chinese Academy of Sciences, Changsha 410125, China

**Keywords:** biomarker, marbling beef, metabonomics, intramuscular fat

## Abstract

**Simple Summary:**

To improve the grade of beef marbling has great economic value in the cattle industry since marbling has the traits of high quality and comprehensive nutrition. This experiment studied the relationship between fecal metabolites and marbling characters, and further screened biomarkers (including ADP, SM(d18:0/16:1(9Z)), PC(15:0/18:2(9Z,12Z)), digoxin, indoleacetaldehyde, PC(16:0/16:0), 3-O-Sulfogalactosylceramide (d18:1/18:0), and spermine).This experiment contributes to a more comprehensive understanding of the molecular mechanism and supplies an essential reference for further exploring the application of metabolite biomarkers to identify marbling beef traits.

**Abstract:**

Background: To improve the grade of beef marbling has great economic value in the cattle industry since marbling has the traits of high quality and comprehensive nutrition. And because of the marbling’s importance and complexity, it is indispensable to explore marbled beef at multiple levels. This experiment studied the relationship between fecal metabolites and marbling characters, and further screened biomarkers. Results: We performed fecal metabolomics analysis on 30 individuals selected from 100 crossbreed cattle (Luxi Yellow cattle ♀ × Japanese Wagyu cattle ♂), 15 with an extremely high-grade marbling beef and 15 with an extremely low-grade marbling beef. A total of 9959 and 8389 m/z features were detected in positive ionization and negative ionization mode by liquid chromatography-mass spectrometry (LC-MS). Unfortunately, the sample separation in the PCA is not obvious, and the predictive ability of the orthogonal partial least squares discrimination analysis (OPLS-DA) model is not good. However, we got six differential metabolites filtered by VIP > 1 and *p* < 0.05. After that, we used weighted correlation network analysis (WGCNA) and found out a module in each positive and negative mode most related to the trait of marbling beef, and then identified three metabolites in positive mode. By further annotation of the Kyoto encyclopedia of genes and genomes (KEGG), it was found that these metabolites involved a variety of metabolic ways, including sphingomyelin metabolism, linoleic acid metabolism, glycerophospholipid metabolism, and so on. Finally, receiver operating characteristic (ROC) analysis was used to evaluate the predictability of metabolites, and the result showed that SM(d18:0/16:1(9Z)) (AUC = 0.72), PC(15:0/18:2(9Z,12Z)) (AUC = 0.72), ADP (AUC = 0.71), PC(16:0/16:0) (AUC = 0.73), and 3-O-Sulfogalactosylceramide (d18:1/18:0) (AUC = 0.69) have an accuracy diagnosis. Conclusions: In conclusion, this study supports new opinions for the successive evaluation of marbling beef through metabolites. Furthermore, six non-invasive fecal metabolites that can evaluate beef marbling grade were found, including SM(d18:0/16:1(9Z)), PC(15:0/18:2(9Z,12Z)), ADP, PC(16:0/16:0), and 3-O-Sulfogalactosylceramide.

## 1. Introduction

Beef marbling, the fat in beef muscle fiber (intramuscular fat, IMF), is distributed in a white marbling shape. It is an important feature that has effects on meat quality and is a valuable factor in beef price. Generally, the more richer the marbles are, the more tender and high-quality the beef is [1,2]. Marbled beef contains a lot of fatty acids needed by the human body. Joo et al. [3] found that highly marbled Hanwoo beef contained a high level of monounsaturated fatty acids (MUFA), which are healthy for the heart due to the ability to lower low-density-cholesterol and increase high-density-cholesterol.

Beef is obtained irreversibly by slaughtering, which may lead to irreversible beef with low meat quality and little marbling. Therefore, it is necessary to find methods to identify beef quality while cattle are alive, to get the better marbling beef through adjusting diets. Non-invasive methods are the better choice. Nowadays, there are a variety of omics methods to explore the molecular mechanism that influences IMF, such as genomics [4,5], transcriptomics [6,7,8], and 16S rRNA gene sequencing [9,10]. However, metabolic profiles are hardly used to explore marbling beef phenotypes. However, metabolites from intestinal microbiota are increasingly regarded as an important factor in human physiology [11]. In recent years, metabolomics has become popular in the research of finding useful new biomarkers [12,13,14]. Fecal metabolites are of great significance as potential biomarkers since they can reflect certain diseases of the large intestine, rectum, and colon, and can especially identify intestinal processes [15]. There have been 6736 described fecal metabolites, including 5.9% of the characterized metabolites [16], indicating that fecal metabolites are potential biomarkers again. In a word, fecal metabonomics analysis provides a non-invasive method to study the relationship between IMF and metabolites to find biomarkers [14]. The gut microbial community plays an important role in animal performance and metabolism [17,18,19,20]. Backhed et al. found that bacteroides can enhance nutrient utilization, break down complex polysaccharides, and thus improve the host’s immune system [21]. In addition, the gut microbiome, such as Oscillospira, Clostridiales, Prevotella, YS2, YS2, and Desulfovibrio have large contributions to the IMF-associated function capacities [22]. In addition, there are substantial associations between several gut microbiota genera and alterations in feces metabolites. For instance, at the FDR = 0.05 threshold, Rashmi et al. found that there were 72 significant metabolite correlations in colorectal cancer patients, including 14 (19%) with Actinobacteria, 15 (21%) with Proteobacteria, 31 (43%) with Firmicutes, 12 (17%) with Bacteroidetes, and none with Fusobacteria or microbes in other phyla [23]. Wu et al. showed that fecal metabolites can also be used to assess feed efficiency in pigs [12]. Therefore, fecal metabolites have the potential to be used to reflect animal health, production performance, and meat quality.

Japanese Wagyu cattle are recognized as the best cattle breed in the world to produce excellent marbling beef [24]. The longissimus lumborum of Japanese Wagyu cattle showed extraordinary amounts of 23.3% IMF at the age of 24 months compared to the IMF of European breeds from 0.6% to 4.7% [25]. Luxi Yellow Cattle is one of the five local cattle breeds in China, and are excellent breeds formed by long-term breeding. The IMF percentage in Luxi Yellow cattle is 4.53 ± 2.58% [26]. We introduced Japanese Wagyu cattle into China, hybridized it with Luxi Yellow cattle of China, and obtained the crossbreed cattle (Luxi Yellow ♀ × Japanese Wagyu ♂)). Luxi Yellow ♀ × Japanese Wagyu ♂ cattle increased the proportion of high-grade marbling beef from 11.85% to 16.62% (The high-grade means grade A3 or above according to Japanese marbling grading standard), and it also increased the carcass weight from 325 to 344 kg [27]. Subsequently, the ideal carcass and meat quality make crossbreed cattle (Luxi Yellow cattle ♀ × Japanese Wagyu cattle ♂) account for increasing sales share in the Chinese beef market. Finally, it is of great significance to further explore the molecular mechanism of intramuscular fat in crossbreed cattle.

## 2. Materials and Methods

The experimental protocol (Permission No. 2020011) was reviewed and approved by the Hunan Agricultural University Institutional Animal Care and Use Committee. The feeding experiment was conducted at Linqing Junbo Food Co., Ltd. (Liaocheng, China) cattle farm.

### 2.1. Experimental Animals and Sample Collection

In this experiment, 30 head of Luxi Yellow×Japanese Wagyu cattle were provided by Linqing Junbo Food Co., Ltd. in Liaocheng, Shandong, China. Accurate 50 mg fecal samples were collected by cryopreservation tube from each cattle at later developmental stage. Before slaughter, the cattle are forbidden to feed or drink water. To discover the fecal samples corresponding to marbling beef grade, after the standardized slaughter in the workshop, 100 g the longissimus lumborum muscle was taken from each cattle and washed with normal saline for scoring according to the Japanese marbling grading standard (beef is divided into grade 1, grade 2, grade 3, grade 4, and grade 5 on the basis of marbling richness. The details are in the Supplementary Figure S1) [6,28,29,30]. Thirty fecal samples were selected, including 15 fecal samples corresponding to low-grade beef (LGB, including grade1–3) and 15 fecal samples corresponding to high-grade beef (HGB, including grade 4–5), then prepared into fecal bacteria liquid, and sent to Majorbio Co., Ltd. in Shanghai, China for LC-MS untargeted metabolomics analysis.

### 2.2. Fecal Sample Pretreatment

Accurate 50 mg fecal samples were weighed and 400 μL cold methanol solution (methanol: water = 4:1, containing 0.02 mg/mL L-2-chlorophenzene alanine) was added into these samples. Subsequently, the mixture was smashed by 50 Hz high-throughput tissue crusher at −10 °C for 6 min. After vortex mixing and ultrasonic extraction for 30 min (5 °C, 40 kHz), the extracted samples stood at −20 °C for 30 min, next centrifuged at 13,000× *g* for 15 min (4 °C), and then the supernatant was drawn and transferred to the injection vial of LC-MS system. In addition, 20 µL of supernatant was removed for each sample and mixed as a quality control sample.

### 2.3. LC-MS Analysis

The UHPLC-Q executive system was provided by ThermoFisher Scientific company for LC-MS analysis. The chromatographic column was an ACQUITY UPLC HSS T3 column (100 mm  × 2.1 mm i.d., 1.8 μm; Waters, Milford, Worcester, MA, USA). The mobile phase A was 95% water: 5% acetonitrile (containing 0.1% formic acid), and the mobile phase B was 47.5% acetonitrile: 47.5% isoprol alcohol: 5% water (containing 0.1% formic acid). The gradient elution procedures were as follows: from 0–0.1 min, 100% (A):0% (B) to 95% (A):5% (B); from 0.1–2.1 min, 95% (A):5% (B) to 75% (A):25% (B); from 2.1–11.1 min, 75% (A):25% (B) to 0% (A):100% (B); 100% (B) maintaining for 4 min; from 15.1–15.2 min, 0% (A):100% (B) to 100% (A):0% (B); 100% (A):0% (B) maintaining for 2.9 min. The flow rate was 0.40 mL/min. The amount of injection was 20 uL and the column temperature was kept at 40 °C. The samples were ionized by electrospray and MS signals were collected in positive and negative ion scanning modes (Spray voltage (+): 3500 V, Spray voltage (—): −2800 V, S-Lens RF Level: 50). The heating temperature and capillary temperature were 400 °C and 320 °C, respectively. The sheath gas flow rate and aux gas flow rate were 40 and 10 arb, respectively. The mass spectrometry scan ranged from 70 to 1050 m/z and the resolution was 17,500 MS^2^.

A QC sample was added every 5–15 samples for the evaluation of analysis system stability.

### 2.4. Data Analysis

The acquired LC-MS data were processed within Progenesis QI (Water Corporation, Milford, Worcester, MA, USA), containing baseline filtering, peak identification, integration, retention time correction, and peak alignment. To get the data matrix used for consecutive analysis, we preprocessed the data as follows: more than 80% of variables with nonzero were saved; missing values were filled by random forests; the Z-score was used for data normalization and only the variables with relative standard deviation <30% of the QC sample were retained.

### 2.5. PCA, PLS-DA, and OPLS-DA Analysis

Before principal component analysis (PCA), partial least squares discrimination analysis (PLS-DA), and orthogonal partial least squares discrimination analysis (OPLS-DA), data were log-transformed, centered, and scaled using the Pareto approach. After preprocessing, the data were subjected to PCA, PLS-DA, and OPLS-DA analysis using the ropls package in R (Version1.6.2) https://www.r-project.org/ (accessed on 2 October 2021) to observe the overall difference between the samples of high and low score marbling samples. The overall contribution of each variable to the PLS-DA model was ranked with variable importance in the projection (VIP), and those metabolites with VIP >1 are considered relevant for marbling beef.

## 3. Results

### 3.1. Fecal Metabolomics Profiling

**Figure 1:**
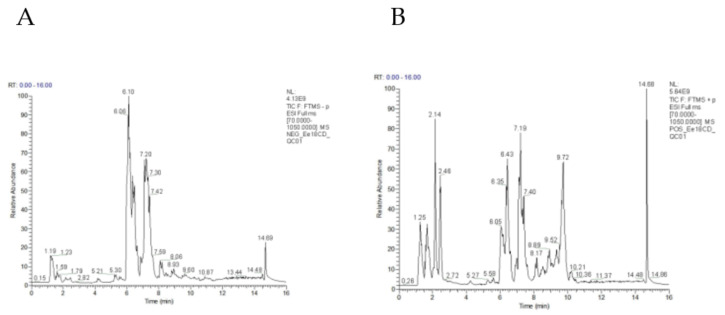
Total ion chromatogram of quality control sample in positive (**A**) and negative (**B**) ion mode.

**Figure 2:**
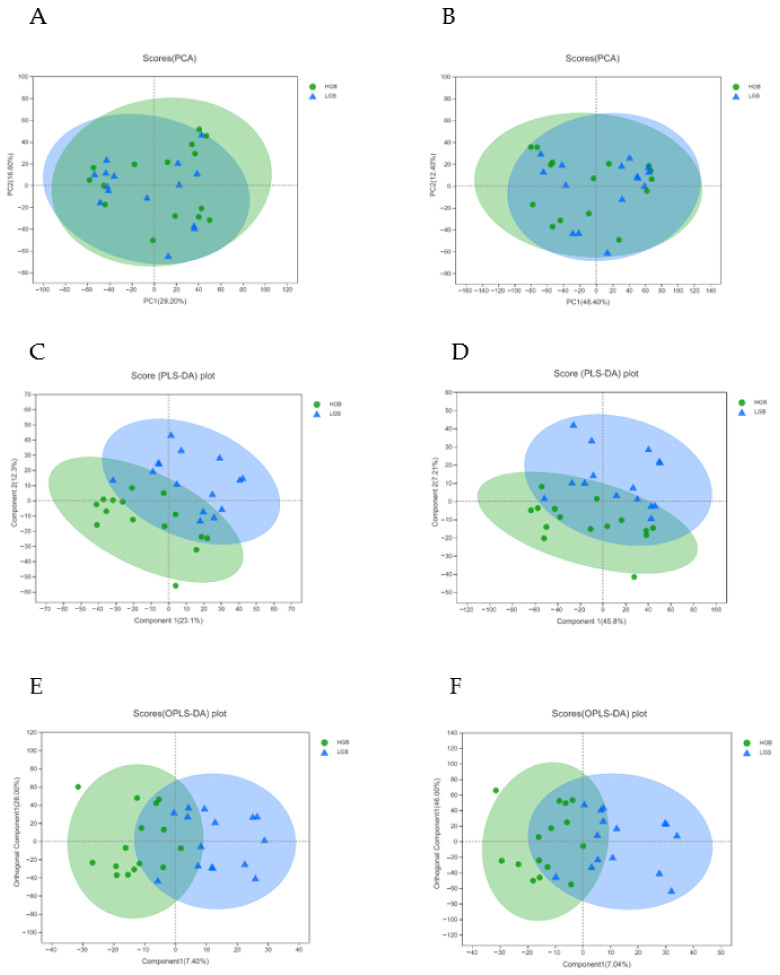
PCA score plots: Principal component analysis of HGB (green) vs. LGB (blue) of positive (**A**) and negative (**B**) using LC-MS data; PLS-DA score plots: partial least squares discrimination analysis of HGB (green) vs. LGB (blue) of positive (**C**) and negative (**D**) using LC-MS data; OPLS-DA score plots: Orthogonal partial least squares discrimination analysis of HGB (green) vs. LGB (blue) of positive (**E**) and negative (**F**) using LC-MS data.

A total of 18,348 peaks (including 9959 positive ions and 8389 negative ions) were obtained from the LC-MS data set (Figure 1A,B). The results of PCA analysis indicated that there was no separation between LGB and HBG groups in positive or negative mode (Figure 2A,B). Moreover, the plots of PLS-DA showed an inapparent separation of LGB vs. HBG (Component 1: R^2^X = 0.231, R^2^Y = 0.275 and Q^2^ = −0.0799 in positive mode; R^2^X = 0.458, R^2^Y = 0.126 and Q^2^ = −0.0169 in negative mode. Component 2: R^2^X = 0.354, R^2^Y = 0.647 and Q^2^ = 0.138 in positive mode; R^2^X = 0.53, R^2^Y = 0.589 and Q^2^ = 0.175 in negative mode.) (Figure 2C,D). Moreover, the results of OPLS-DA also showed a separation of LGB vs. HBG (Sum of component 1 and orthogonal component 1: R^2^X = 0.354, R^2^Y = 0.647 and Q^2^ = −0.137 in positive mode; R^2^X = 0.53, R^2^Y = 0.589 and Q^2^ = −0.177 in negative mode) (Figure 2E,F), and a low Q^2^ value (Q^2^ < 0.5) indicated poor predictability.

### 3.2. Differential Metabolite from OPLS-DA Analysis

**Figure 3:**
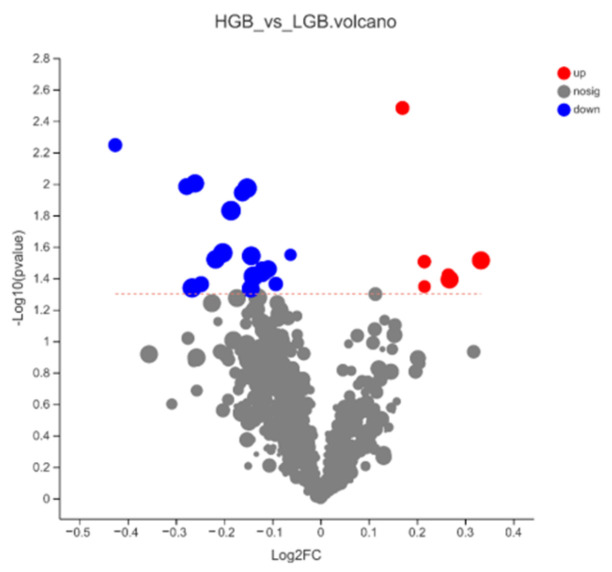
Differential metabolite analysis. Volcano plot: the abscissa is the multiple change value of metabolite expression difference of HGB vs. LGB, and the ordinate is the statistical test value of metabolite expression difference. The higher the ordinate value, the more significant the expression difference is. Each point in the figure represents a specific metabolite, and the size of the point represents the VIP value.

However, a total of 29 differential metabolites (18 positive ions and 9 negative ions) with important contributions (*p* < 0.05, VIP from OPLS-DA analysis > 1) were screened using Student’s *t*-test (Unpaired) and two-tailed test (Table S1). According to HMDB annotation, there were fifteen lipids or lipid-like molecules, three organic oxygen compounds, three organoheterocyclic compounds, two benzenoids, one organic acid or derivative, one nucleoside or analogue, one organic nitrogen compound, and one unannotated compound (Table S1). Compared with LGB, downregulated metabolites (VIP > 1) of HGB were more than upregulated metabolites (Figure 3). After KEGG annotation, we found six metabolites, and the pathways can be divided into five classes, including metabolism (global and overview maps, nucleotide metabolism, lipid metabolism, metabolism of terpenoids and polyketides, amino acid metabolism, and energy metabolism), human diseases, environmental information processing, cellular processes and organismal system (Table S1). To be specific, SM(d18:0/16:1(9Z)) and PC(15:0/18:2(9Z,12Z)) correlated with marbling standard longissimus lumborum are related to lipid metabolism. Spermine is involved in amino acid metabolism, which is also related to the phenotype of marbling beef. The remaining three are ADP, indoleacetaldehyde, and digoxin (Table 1).

### 3.3. Differential Metabolite from WGCNA Analysis

**Figure 4:**
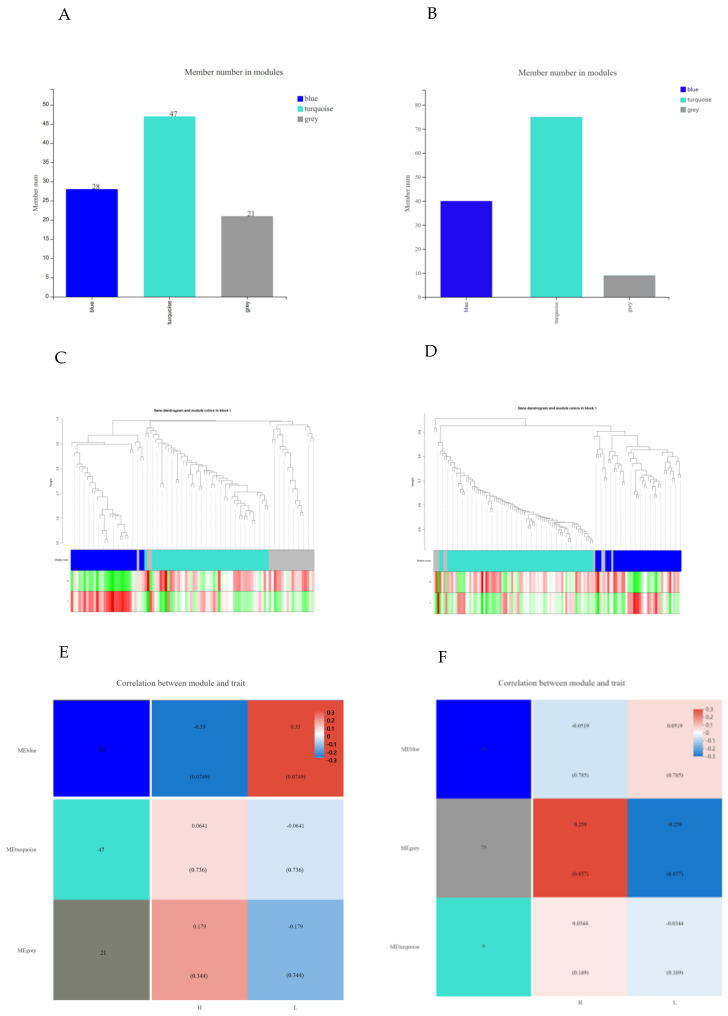
Weighted correlation network analysis (WGCNA). Member number in modules of positive (**A**) and negative (**B**) modes. Cluster dendrogram and modular trait correlation diagram in positive (**C**) and negative (**D**) modes; green represents downregulation and red represents upregulation. Heat map of correlation between metabolites and phenotype in positive (**E**) and negative (**F**) modes. Each color line represents a module.

WGCNA analysis was carried out due to unsatisfactory PCA and OPLS-DA analysis models. WGCNA analysis is an approach to gathering genes with similar characteristics into a module and finding hub genes from the hub module. In this experiment, WGCNA was used for investigating the corporation between hub metabolites and phenotypes. After merging modules with similar features (Supplementary Figure S2A,B), a total of three modules were obtained in positive (negative) mode (Figure 4A,B), and there were 28 (40) features in the the MEblue module, 47 (75) features in turquoise module, and 21 (9) features in grey module. Next, through calculating the correlation between module and phenotype concerning IMF, we found the module most related to the sample phenotype. As shown in Figure 4E,F, metabolites in the MEblue module of positive mode had the closest correlation with the phenotype of IMF (R = 0.33, *p* = 0.0749). Equally, metabolites in the MEgrey module of negative mode are most related to the phenotype (R = 0.258, *p* = 0.169). However, the MEgrey module in WGCNA has no meaning, and the metabolites in the MEgrey module are not recommended to be a biomarker. For detailed information about metabolites in the MEblue module from positive mode, see Table S1. Then, by KEGG annotation, we found three metabolites in the MEblue module of positive mode. There were three pathways involved in the three metabolites, including metabolism (global and overview maps, lipid metabolism, metabolism of other amino, amino acid metabolism, metabolism of other amino acids, and metabolism of cofactors and vitamins), human diseases (cancer: overview), and organismal systems (nervous system, digestive system). Separately, PC(16:0/16:0) is related to metabolic pathways (map01100), glycerophospholipid metabolism (map00564), biosynthesis of secondary metabolites (map01110), arachidonic acid metabolism (map00590), linoleic acid metabolism (map00591), alpha-Linolenic acid metabolism (map00592), choline metabolism in cancer (map05231), and retrograde endocannabinoid signaling (map04723). 3-O-Sulfogalactosylceramide (d18:1/18:0) is related to sphingolipid metabolism (map00600) and metabolic pathways (map01100). Spermine is related to metabolic pathways (map01100), bile secretion (map04976), glutathione metabolism (map00480), arginine and proline metabolism (map00330), and beta-Alanine metabolism (map00410). The above three metabolites in the positive ion mode were all negatively correlated with the IMF. Through calculation, the connectivity of metabolites in the module was obtained (Table 2).

### 3.4. ROC Analysis

**Figure 5:**
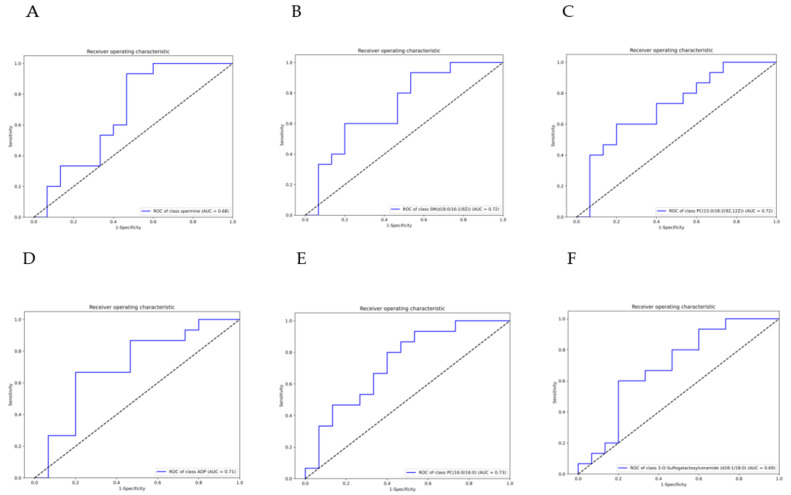
Receiver operating characteristic (ROC) analysis of spermine (**A**) (AUC = 0.68), SM(d18:0/16:1(9Z)), (**B**) (AUC = 0.72), PC(15:0/18:2(9Z,12Z)), (**C**) (AUC = 0.72), ADP (**D**) (AUC = 0.71), PC(16:0/16:0), (**E**) (AUC = 0.73), and 3-O-Sulfogalactosylceramide (d18:1/18:0) (**F**) (AUC = 0.69). Area Under Curve (AUC) values of ROC analysis are usually between 1.0 and 0.5. In the case of AUC > 0.5, the closer the AUC is to 1, the better the diagnosis. AUC has an accuracy at 0.5 to 0.7, a certain accuracy at 0.7 to 0.9, and a higher accuracy when it is above 0.9. AUC ≤ 0.5 indicates that the diagnostic method is completely ineffective and has no diagnostic value.

From the results of the WGCNA analysis and differential metabolite analysis, there is only one metabolite, spermine, belonging to both the two analysis methods. To explore the quality of this metabolic, we did receiver operating characteristic (ROC) analysis and drew a ROC curve to show the sensitivity and specificity. We found that the model has good prognostic effectiveness (Area Under Curve (AUC) of spermine = 0.68). We also did ROC analysis for the two differential metabolites closely related to fat metabolism, and obtained the results AUC of SM(d18:0/16:1(9Z)) = 0.72, PC(15:0/18:2(9Z,12Z)) = 0.72, ADP (AUC = 0.71), PC(16:0/16:0) (AUC = 0.73), and 3-O-Sulfogalactosylceramide (d18:1/18:0) (AUC = 0.69). (Figure 5A–F).

## 4. Discussion

With the improvement in people’s economic level, the demand for meat quality is becoming higher and higher, followed by the trend of high-grade marbling beef in the market. Therefore, improving marbling traits in beef is of significant economic meaning. Any process that can effectively predict the marbling grade of beef is of great significance for meat production. It is complicated to estimate marbling traits of beef as animals are alive. Although there are currently numerous studies on marbling at the molecular level, most of them are related to the biological pathway and regulatory factors of the marbling level [6,28,31], and few studies have explored molecular metabolites as a biomarker to estimate marbling phenotypic traits. In our study, we used the technology of LC-MS and the analysis method of PCA, OPLS-DA, WGCNA, and ROC to compare and analyze fecal metabolites in HGB and LGB groups.

In this study, we used two methods to analyze the quantitative trait, namely PCA and OPLS-DA and WGCNA. One method, PCA and OPLS-DA, gathers quantitative traits based on threshold. To be specific, this method directly divides experimental animals into a high or low grade of marbling beef for analysis. This analysis method can seek out the impact factors that affect phenotypes with large weight quickly. Another method, WGCNA, directly associates the value of quantitative traits with impact factors. It can more comprehensively consider the impact of the continuity of metabolite changes on the phenotype. These two methods play a complementary role and help to find the factors affecting marbling traits more quickly and comprehensively.

**Figure 6:**
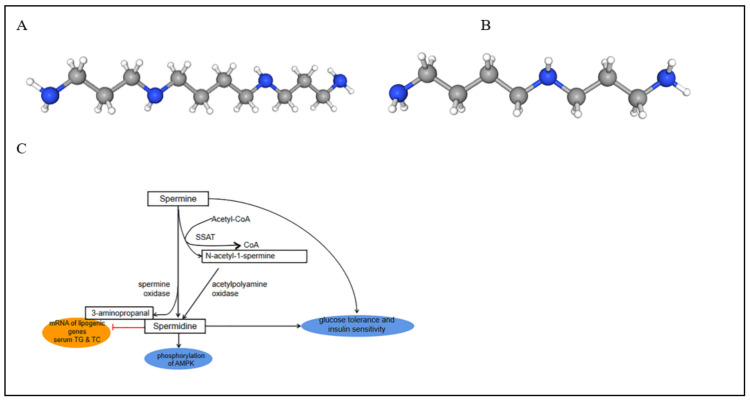
Molecular structure of spermine (**A**) and spermidine (**B**). 
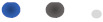
: N, C, H, and their metabolic pathways (**C**).

We obtained differential metabolites from OPLS-DA (VIP > 1, *p* < 0.5), and found the hub metabolites from the module most similar to the phenotype through WGCNA. Notably, although differential metabolites from OPLS-DA and WGCNA were obtained, the model of OPLS-DA has poor predictability, and the differences in WGCNA are not very significant. Through a comprehensive analysis of the two methods, we obtained the most representative biomarker, spermine (*p* = 0.043, VIP = 2.121 and the correlation of metabolite and trait = −0.239) (Figure 6A, Table S1 and Table 2). Spermine is the most basic polyamine. Monelli et al. [32] reported that humans and mice with obesity had low polyamine levels in white adipose tissue, which may be explained by the hypothesis: acetyl CoA is a substrate for acetyl CoA carboxylase, which generates an intermediate used for fatty acid synthesis malonyl-CoA. Polyamines activate both lipolysis and fatty acid oxidation in white adipose tissue, and the polyamine flux and catabolism consume acetyl CoA and cellular energy, resulting in an ineffective cycle that consumes white adipose tissue and accelerates glucose and lipid metabolism [33,34]. Interestingly, excessive depletion of polyamine would lead to lipid reduction by suppressing the key regulators expression of adipocyte differentiation, such as peroxisome proliferator-activated receptor γ (PPARγ) and CCAAT/enhancer-binding protein α (C/EBPα) [35]. Moreover, spermine has a function similar to insulin [36]. The study by Satish et al. showed that mice with spermine treatment at a dose of 10 mg/kg resulted in a 24% reduction in body weight and an 18% reduction in fasting glucose [33]. Furthermore, as the decomposition product of spermine, spermidine has been well-known to be negatively correlated with lipid accumulation [37,38]. There are two pathways that convert spermine to spermidine (Figure 6B,C), including spermine oxidase which converts spermine into spermidine and 3-aminopropanal and acetylpolyamine oxidase, which converts spermine into spermidine and N-acetyl-1-spermine [39]. After the generation of spermidine, it can ameliorate non-alcoholic fatty liver disease by regulating lipid metabolism, and spermidine also inhibits the expression of lipogenic genes dependent on adenosine monophosphate-activated protein kinase (AMPK) [40]. To be specific, the administration of spermidine can inhibit the lipogenesis process by increasing the phosphorylation of AMPK and simultaneously decreasing the mRNA levels of lipogenic genes in mouse primary hepatocytes [40]. Spermidine supplementation can enhance glucose tolerance and insulin sensitivity [41], and can also decrease the mean diameter of adipocytes, hepatosteatosis, serum total cholesterol (TC), and triglycerides (TG) [42,43]. The metabolism of spermine and spermidine plays an important role in fat metabolism and the phenotype of marbling beef. The WGCNA analysis in our results showed that there was a significant negative correlation between the metabolite spermine and the phenotype of high-grade marbling beef. This present result was consistent with the research of predecessors. In our study, ROC analysis was used for the preliminary evaluation of the biomarkers. The result showed that metabolite spermine as a biomarker has good prediction ability. In a word, spermine has eminent potential to be widely used in cattle husbandry after further rigorous assessment. Notably, in the process of polyamine metabolism, the downstream metabolite spermidine has no significant difference between the HGB and LGB groups. We hope that with the duration improvement of the metabonomics database, there will be enough literature in the future to explain it.

**Figure 7:**
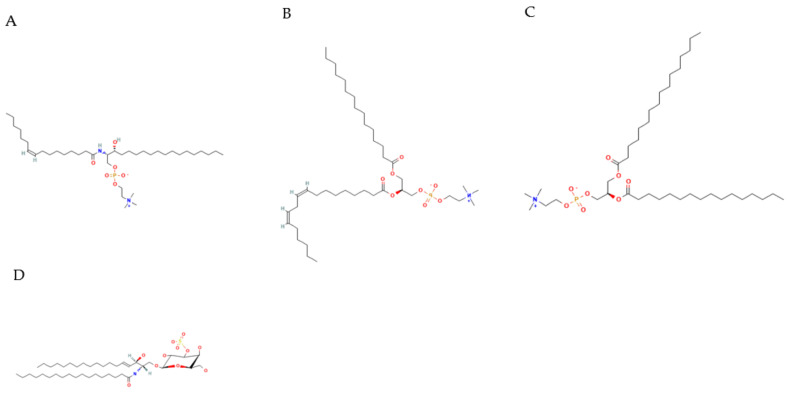
Molecular structure of SM(d18:0/16:1(9Z)) (**A**), PC(15:0/18:2(9Z,12Z)) (**B**), PC(16:0/16:0) (**C**), and 3-O-Sulfogalactosylceramide (d18:1/18:0) (**D**).

For other differential metabolites from OPLS-DA analysis, SM(d18:0/16:1(9Z)) (*p* = 0.027, VIP = 3.136) (Figure 7A), ADP (*p* = 0.046, VIP = 3.043) and PC(15:0/18:2(9Z,12Z)) (Figure 7B) are also closely related to fat metabolism. SM(d18:0/16:1(9Z)) is a kind of sphingomyelin correlated with sphingomyelin metabolism. A study reported that ratios between sphingomyelins (SM) and phosphatidylcholines were increased in obese children compared to normal-weight children [44]. In addition, SM (d18:0/16:1(9Z)) in rats was positively correlated with total cholesterol, TG, low-density lipoprotein cholesterol, and high-density lipoprotein cholesterol [45]. However, most research has proven that SM inhibits lipid absorption in rodents [46,47,48]. In our experiment, SM (d18:0/16:1(9Z)) is negatively correlated with marbling beef and intramuscular fat, which is similar to most research. In terms of Adenosine-5′-diphosphate (ADP), ADP is a purine ribonucleoside 5′-diphosphate, having adenine as the nucleobase. It is a fundamental metabolite in organisms [49]. Through ATP synthase, ADP can be converted into ATP and directly provide energy for organisms. In the human body, ADP is converted to AMP (AMP kinase), AMP is converted to IMP (AMP deaminase), IMP is hydrolyzed to inosine (5′-nucleotidase), inosine is phosphorylated to hypoxanthine (nucleoside phosphorylase), hypoxanthine is oxidized to xanthine (xanthine oxidase), and xanthine is oxidized to uric acid (xanthine oxidase), which is excreted from the body with urine. Interestingly, consumption of a high-fat (HF) diet increases the content of mitochondrial, but impairs mitochondrial ADP sensitivity in skeletal muscle of mice and decreases the concentration of ADP [50]. The specific process may be that, after an HF diet, impairment of ATP synthase still exists and the HF diet also decreases the sensitivity of adenine nucleotide translocase (ANT) to ADP binding; meanwhile, the decreased sensitivity is likely amplified by increases in intramuscular P-CoA concentrations [51,52]. This may also be the reason that the concentration of ADP in the HGB group is lower than in the LGB group in our experiment. As for the metabolite of PC(15:0/18:2(9Z,12Z)), it belongs to phosphatidylcholines (PC), which are a class of phospholipids that incorporate a phosphocholine headgroup into a diacylglycerol backbone. They are the most abundant phospholipid in eukaryotic cell membranes and have both structural and signaling roles [53]. In addition, PCs are related to anti-inflammation mechanisms, cholesterol metabolism, neuronal differentiation, mitochondrial function, and insulin resistance [54,55,56]. A study by Inoue et al. [57] showed that changes to the carbohydrate–fat ratio in a diet greatly affect plasma PC profiles; the study also found biomarkers reflecting the dietary–fat ratio, such as (PC(16:0/16:1) and PC(16:0/18:1)). Many studies have provided a hypothesis that PC can reduce fat deposition [58,59,60]. Lee et al. [60] found that PC restrained body weight gain and lipid accumulation and alleviated hyperlipidemia by lessening TG and TC levels and enhancing the HDL-TC ratio. Similarly, our result that PC(15:0/18:2(9Z,12Z)) negatively correlated with marbling beef traits was consistent with the effect of PC on lipid metabolism. Digoxin is often used as a drug for heart disease [61,62]. Indoleacetaldehyde is usually regarded as a growth hormone in higher plants [63]. However, there is rare literature on fat metabolism related to the above two metabolites.

The other two metabolites from WGCNA analysis are PC(16:0/16:0) (Figure 7C) and 3-O-Sulfogalactosylceramide (d18:1/18:0) (Figure 7D). The function of PC(16:0/16:0) is consistent with PC(15:0/18:2(9Z,12Z)), which is also negatively correlated with the trait of marbling beef in our experiment. The metabolite 3-O-Sulfogalactosylceramide (d18:1/18:0) is a kind of sulfatide, which is closely related to sphingolipid metabolism [64], and it also has the function of anti-inflammation by inhibiting the production of IL-1, IL-6, IL-10, and TNF-α [65] or by hindering the co-localization of TLR4 and lipid rafts and nullifying the effect of LPS on TLR4 signaling [66]. Moreover, sulfatide cures type 2 diabetes through the activation of potassium channels [67]. As is known, changes in fat mass were directly connected with inflammation factors [68]. Hence, the relationship between sulfatide and fat mass could be negatively correlated. This may be the reason that 3-O-Sulfogalactosylceramide (d18:1/18:0) is negatively associated with intramuscular fat and marbling beef traits. Based on our experiment, these findings can also provide some references for further analysis of fat metabolism.

## 5. Conclusions

The present study focused on finding biomarkers according to the differential fecal metabolites of high or low-grade marbling meat cattle. We obtained six metabolites by OPLS-DA analysis (VIP > 1, *p* < 0.05), namely ADP, SM(d18:0/16:1(9Z)), PC(15:0/18:2(9Z,12Z)), digoxin, indoleacetaldehyde, and spermine. However, the model of PCA and OPLS-DA has low explanatory variances for the phenotypes of marbling beef and intramuscular fat. Hence, we used WGCNA co-expression analysis and found four metabolites from two essential modules related to traits in the positive and negative modes, namely PC(16:0/16:0), 3-O-Sulfogalactosylceramide (d18:1/18:0), and spermine. An ROC model was used to evaluate the metabolites above, which provides the possibility of collecting the metabolic marks in the feces in the future. In comprehensive consideration, spermine is the most worthy biological marker for exploring. Overall, this study identified candidate biomarkers closely related to marbling beef (SM(d18:0/16:1(9Z)), PC(15:0/18:2(9Z,12Z)), ADP, PC(16:0/16:0), and 3-O-Sulfogalactosylceramide), which contributes to a more comprehensive understanding of the molecular mechanism and supplies an essential reference for further exploring the application of metabolite biomarkers to identify marbling beef traits.

## Figures and Tables

**Table 1:** The six metabolites in the positive and negative modes obtained by Student’s *t*-test and KEGG pathway annotation.

Metabolite	Formula	VIP_Pred_OPLS-DA	FC(HGB/LGB)	*p*-Value	FDR	Mode	M/Z
ADP	C10H15N5O10P2	3.043	0.831	0.046	0.837	neg	426.021
SM(d18:0/16:1(9Z))	C39H79N2O6P	3.136	0.869	0.027	0.896	pos	725.556
PC(15:0/18:2(9Z,12Z))	C41H78NO8P	2.907	0.905	0.029	0.896	pos	766.536
Digoxin	C41H64O14	2.731	0.835	0.010	0.896	pos	745.420
Indoleacetaldehyde	C10H9NO	1.749	0.938	0.043	0.856	pos	160.076
Spermine	C10H26N4	2.121	0.842	0.043	0.896	pos	203.223

Note: Abbreviations: FC, fold-change. VIP value, The variable importance in projection in OPLS-DA analysis. FDR, False discovery rate.

**Table 2:** The three metabolites in the MEblue module of positive mode obtained by KEGG pathway annotation.

Metabolite	M/Z	Mode	Metabolite and Trait Correlation	Formula
PC(16:0/16:0)	756.551	pos	−0.361	C40H80NO8P
3-O-Sulfogalactosylceramide (d18:1/18:0)	846.522	pos	−0.359	C42H81NO11S
Spermine	203.223	pos	−0.239	C10H26N4

## Data Availability

The data presented in this study are available in the manuscript.

## Supplementary Materials

The following supporting information can be downloaded at: https://www.mdpi.com/article/10.3390/ani12172243/s1, Figure S1: The official Japanese Meat Grading Standards; Figure S2: Scale independence and mean connectivity of various soft thresholds. Table S1: The detailed information of differential metabolites.
