# Supplementary material for: Using Untargeted LC-MS Metabolomics to Identify the Association of Biomarkers in Cattle Feces with Marbling Standard Longissimus Lumborum
Source: Animals (Basel). 2022 Aug 30;12(17):2243. doi: 10.3390/ani12172243 (PMC9455031; doi:10.3390/ani12172243)
Supplement: Supplementary file 1 [file animals-12-02243-s001.zip › animals-1832516-supplementary/Figures S1 and S2.pdf]

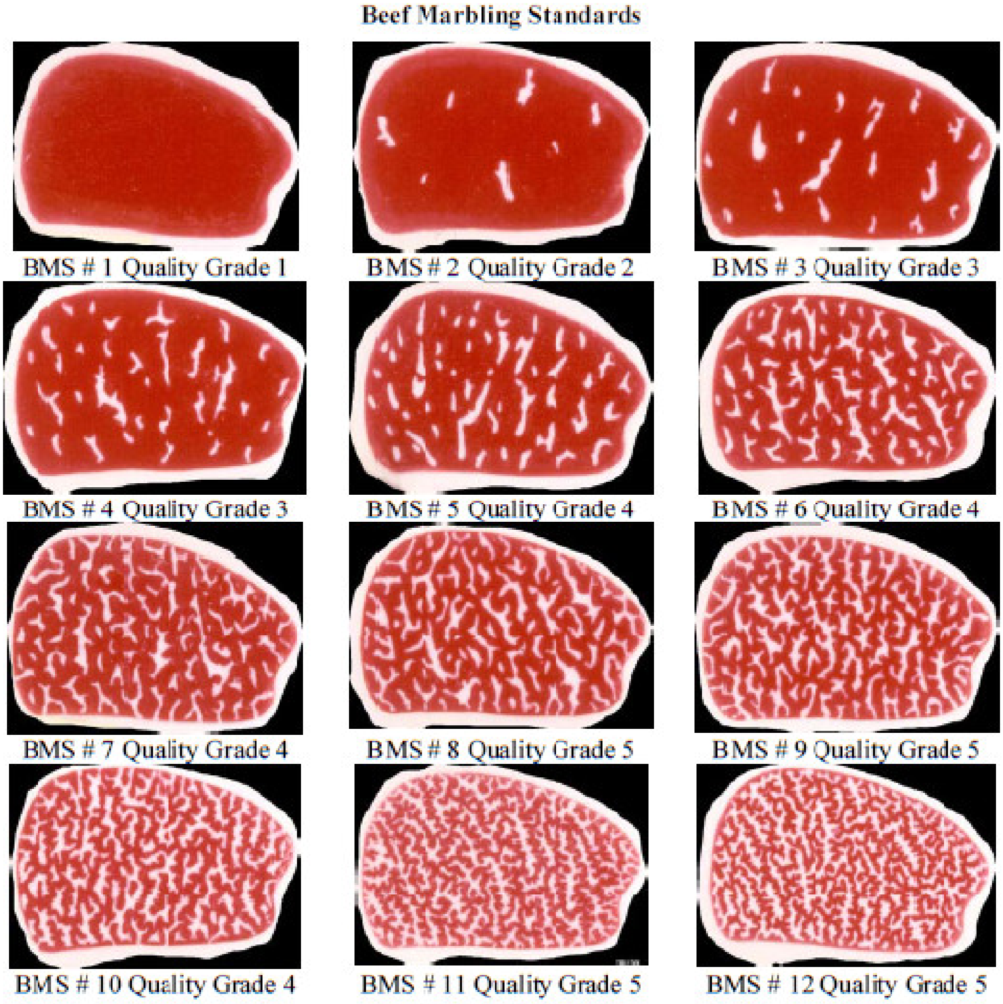

Figure S1. The official Japanese Meat Grading Standards which have been adopted by the Chinese beef cattle industry along with other meat grading standards.

A

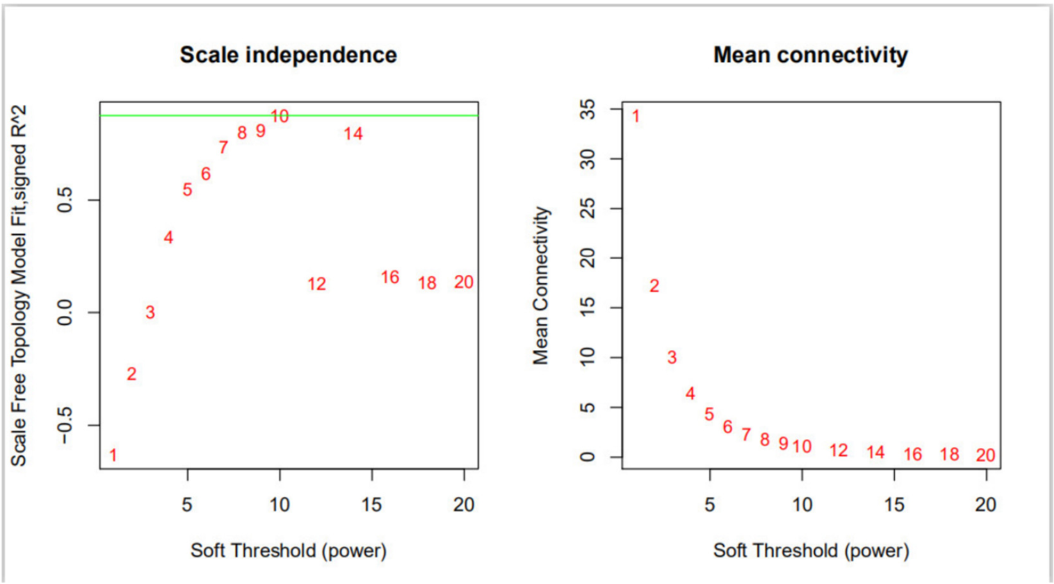

B

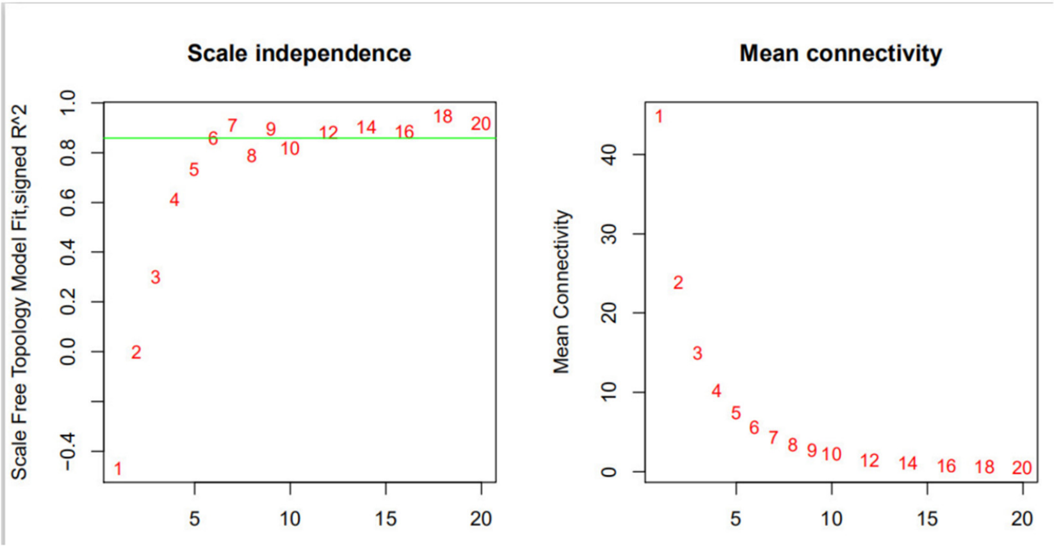

Figure S2. Scale independence and mean connectivity of various soft thresholds in positive mode (**A**) and negative mode (**B**). Left: the abscissa represents the soft threshold, and the ordinate represents the evaluation parameter R^2 of the scale-free network. The higher the value, the more consistent with the scale-free characteristics. Right: the abscissa represents the soft threshold and the ordinate represents the average connectivity. Generally, the soft threshold when the correlation between soft threshold and scale-free topology reaches 0.85 is required as β Value, you can see that in this example β = 10 (in positive mode) and β = 6 (in negative mode), get β Value, according to β Value to obtain the proximity matrix and topological matrix.
